# PRV UL13 inhibits cGAS–STING-mediated IFN-β production by phosphorylating IRF3

**DOI:** 10.1186/s13567-020-00843-4

**Published:** 2020-09-15

**Authors:** Zongyi Bo, Yurun Miao, Rui Xi, Qiuping Zhong, Chenyi Bao, Huan Chen, Liumei Sun, Yingjuan Qian, Yong-Sam Jung, Jianjun Dai

**Affiliations:** MOE Joint International Research Laboratory of Animal Health and Food Safety, MOA Key Laboratory of Animal Bacteriology, College of Veterinary Medicine, Nanjing Agricultural University, Nanjing, 210095 China

**Keywords:** pseudorabies virus, cGAS–STING, UL13 serine/threonine kinase protein, IRF3

## Abstract

Cyclic GMP-AMP (cGAMP) synthase (cGAS) is an intracellular sensor of cytoplasmic viral DNA created during virus infection, which subsequently activates the stimulator of interferon gene (STING)-dependent type I interferon response to eliminate pathogens. In contrast, viruses have developed different strategies to modulate this signalling pathway. Pseudorabies virus (PRV), an alphaherpesvirus, is the causative agent of Aujeszky’s disease (AD), a notable disease that causes substantial economic loss to the swine industry globally. Previous reports have shown that PRV infection induces cGAS-dependent IFN-β production, conversely hydrolysing cGAMP, a second messenger synthesized by cGAS, and attenuates PRV-induced IRF3 activation and IFN-β secretion. However, it is not clear whether PRV open reading frames (ORFs) modulate the cGAS–STING-IRF3 pathway. Here, 50 PRV ORFs were screened, showing that PRV UL13 serine/threonine kinase blocks the cGAS–STING-IRF3-, poly(I:C)- or VSV-mediated transcriptional activation of the IFN-β gene. Importantly, it was discovered that UL13 phosphorylates IRF3, and its kinase activity is indispensable for such an inhibitory effect. Moreover, UL13 does not affect IRF3 dimerization, nuclear translocation or association with CREB-binding protein (CBP) but attenuates the binding of IRF3 to the IRF3-responsive promoter. Consistent with this, it was discovered that UL13 inhibits the expression of multiple interferon-stimulated genes (ISGs) induced by cGAS–STING or poly(I:C). Finally, it was determined that PRV infection can activate IRF3 by recruiting it to the nucleus, and PRVΔUL13 mutants enhance the transactivation level of the IFN-β gene. Taken together, the data from the present study demonstrated that PRV UL13 inhibits cGAS–STING-mediated IFN-β production by phosphorylating IRF3.

## Introduction

During viral infection, host cells recognize pathogen-associated molecular patterns (PAMPs) or damage-associated molecular patterns (DAMPs) via pattern recognition receptors (PRRs) and elicit downstream cascades of antiviral innate immune responses [1–3]. Cyclic GMP-AMP (cGAMP) synthase (cGAS) has been identified as a cytosolic DNA sensor and plays an important role in type I interferon responses. Upon sensing pathogen DNA, cGAS catalyses the synthesis of cGAMP, a second messenger that subsequently activates stimulator of interferon genes (STING), TANK-binding kinase 1 (TBK1), and interferon response factor 3 (IRF3) or NF-κB signal pathways to promote the production of interferon-β (IFN-β) [4–7].

To counteract the antiviral effect of cGAS–STING signalling, viruses have evolved to different evasion strategies. Herpesviruses, a group of large dsDNA viruses, have been intensively studied [8–10]. For instance, Kaposi’s sarcoma-associated herpesvirus (KSHV) encodes the ORF52 tegument protein and latency-associated nuclear antigen (LANA), which inhibit cGAS enzymatic activity via cGAS and/or DNA binding [11]. KSHV vIRF1 not only blocks the phosphorylation of STING by preventing its interaction with TBK1 but also interrupts p300/IRF3 complex formation or inhibits p300 activity [12, 13]. Herpes simplex virus 1 (HSV-1) VP24 and ICP27 target the interaction between TBK1 and IRF3 and the TBK1-STING signalosome, respectively, impairing IRF3 activation [14, 15]. HSV-1 UL24 binds to the endogenous NF-κB subunits p65 and p50 and selectively blocks NF-κB but not IRF3 promoter activation [16]. Moreover, Marek’s disease virus (MDV) VP23 inhibits the DNA-sensing pathway by suppressing IRF7 activation [17]. The MDV major oncoprotein Meq impedes the recruitment of TBK1 and IRF7 to the STING complex and inhibits IRF7 activation and IFN-β induction [18].

Pseudorabies virus (PRV), also called suid herpesvirus 1 (SuHV-1) or Aujeszky’s disease virus (ADV), is a member of the family *Herpesviridae*, subfamily *Alphaherpesvirinae*, and genus *Varicellovirus*. PRV causes considerable damage to the swine industry in many countries. In addition to swine, its natural reservoir, PRV can also infect a broad spectrum of mammals, including cattle, sheep, canines, foxes, wolves, and tigers. In addition, PRV preferentially infects the peripheral nervous system [19]. Therefore, research on PRV bears significance for disease control in the swine industry; PRV can be used as a model for herpesvirus research and as a live tracer of neuronal circuitry.

To facilitate its replication in hosts, PRV has developed multiple strategies for evading the host defence system. For instance, cDNA microarray analysis indicates that a subset of interferon-stimulated genes (ISGs) stimulated by IFN-β in primary rat fibroblast cells is suppressed in cells infected with PRV [20]. PRV US3 protein can prevent cell apoptosis during infection and treatment with sorbitol or staurosporine [21]. PRV EP0 can counteract the interferon-mediated antiviral response in primary cells isolated from the natural host of PRV [22]. PRV UL49.5 acts as a transporter associated with antigen processing (TAP) inhibitor, impeding the presentation of virus-derived peptides by using major histocompatibility complex (MHC) class I molecules to prevent the recognition and elimination of virus-infected cells by cytotoxic T lymphocytes [23]. PRV UL50 can suppress type-I IFN signalling by promoting the lysosomal degradation of IFNAR1 [24]. PRV US3 impairs IFN-mediated antiviral activity by degrading Bcl-2 associated transcription factor 1 (Bclaf1), a positive regulator in IFN signalling [25].

In the current study, we explored how PRV UL13 regulates the cGAS–STING pathway. We demonstrated that PRV UL13, a serine/threonine kinase protein, inhibits cGAS–STING-mediated IFN-β production via IRF3 phosphorylation. On the other hand, PRV UL13 exerts no effect on IRF3 dimer formation, nuclear translocation, or association with CBP; it inhibits the recruitment of activated IRF3 to the IRF3-responsive promoter and subsequent expression of ISGs induced by the cGAS–STING pathway and polyinosinic:polycytidylic acid (poly[I:C]). Accordingly, UL13-deficient PRV enhances IFN-β transactivation in PK15 cells. To our knowledge, this study is the first to show that PRV UL13 can block IFN-β production mediated by the cGAS–STING pathway.

## Materials and methods

### Cells and viruses

PK15, 293T, A549 and MDCK cells were purchased from ATCC. Vero-E6 was generously provided by Dr. Fei Liu from the College of Veterinary Medicine, Nanjing Agricultural University. The cells were cultured with Dulbecco’s modified Eagle medium (DMEM, GIBCO) supplemented with 8% fetal bovine serum (Pan Biotech UK, Ltd., Dorset, UK) and a 1% penicillin–streptomycin solution (Beyotime Biotechnology, Shanghai, China) at 37 ℃ in a 5% CO_2_ incubator. The PRV field strain JSY13 (MT157263.1) was isolated and stored in our laboratory. The vesicular stomatitis virus (VSV) was stored in our laboratory.

### Plasmids

PRV ORFs amplified from the PRV genome, as well as pig STING (NM_001142838.1) amplified from the cDNA of PK15 cells, were cloned into pcDNA3-Flag. UL13 kinase-dead mutants (UL13-K103A, UL13-D194A/V195A, UL13-N199A/I200A, and UL13-D216A) were constructed by replacing essential kinase activity sites with alanine (A) via site-directed mutagenesis. UL13-K103A was generated with the sense primer, 5′-TGT ACG GCT CGG TGG CCG TGG CAA CGC TCC GCG CCG-3′, and the antisense primer, 5′- GAA GCC GGC GCG GAG CGT TGC CAC GGC CAC CGA-3′. UL13-D194A/V195A was generated with the sense primer, 5′-GCG GGC TCA GCC ACC TGG CCG CCA AGG GCG GCA ACA-3′, and the antisense primer, 5′-GAT GTT GCC GCC CTT GGC GGC CAG GTG GCT GAG CCC G-3′. UL13-N199A/I200A was generated with the sense primer, 5′-ACG TCA AGG GCG GCG CAG CAT TTG TGC GCA CGT G-3′, and the antisense primer, 5′-CAC GTG CGC ACA AAT GCT GCG CCG CCC TTG ACG TC-3′. UL13-D216A was generated with the sense primer, 5′-CGG CCG TCA TCG GGG CCT TTA GCC TCA TGG CCC-3′, and the antisense primer, 5′-GGG CCA TGA GGC TAA AGG CCC CGA TGA CGG CCG-3′. Pig cGAS (XM_013985148.2), TBK1 (XM_021090852.1), and IRF3 (NM_213770.1) amplified from the cDNA of PK15 cells were cloned into pcDNA4-HA. IRF3/5D was generated using a method described in a previous study [26]. The luciferase reporter plasmids used in this study were IFN-β-Luc and IRF3-Luc; the former contains the − 296 to + 52 fragment of the pig IFN-β promoter, and the latter contains four copies of the IRF3-binding positive regulatory domain [27].

### Antibodies and reagents

Mouse anti-Flag, rabbit anti-actin, horseradish peroxidase (HRP)-conjugated goat anti-mouse IgG (Fab) and HRP-conjugated goat anti-rabbit IgG (Fab) were supplied by Sigma; the mouse anti-HA was provided by Abcam; rabbit anti-IRF3 was purchased from Proteintech; mouse anti-CBP was supplied by Santa Cruz Biotechnology. The following antibodies were purchased from Millipore: HRP-conjugated goat anti-mouse IgG (H + L), HRP conjugated goat anti-rabbit IgG (H + L), Alexa Fluor 555-conjugated goat anti-mouse IgG antibody, Alexa Fluor 555-conjugated goat anti-rabbit IgG antibody, Alexa Fluor 488 conjugated goat anti-mouse IgG antibody, and Alexa Fluor 488-conjugated goat anti-rabbit IgG antibody. Lambda protein phosphatase was supplied by New England Biolabs; poly(I:C) was provided by Invitrogen; CCK-8 was purchased from ApexBio.; and ECL was supplied by Thermo Fisher Scientific.

### Dual-luciferase reporter assay

The dual-luciferase reporter assay was performed in triplicate in accordance with the instructions provided by the manufacturer (Promega). PK15 cells were seeded at 6 × 10^4^ cells per well in 24-well plates overnight and then transfected with the indicated plasmids using Lipofectamine 2000 reagent (Invitrogen) in accordance with the manufacturer’s instructions to screen the PRV ORFs that may modulate the cGAS–STING-induced transactivation of the IFN-β gene promoter, as well as to evaluate the effect of UL13 on the transactivation of the IFN-β promoter by the cGAS–STING effectors, TBK1, and IRF3. At 30 h post transfection, the cells were harvested to analyse firefly and Renilla luciferase activity using a dual-luciferase assay kit (Promega). Approximately 24 h post-transfection, the PK15 cells were treated with poly(I:C) (10 μg/mL) or infected with VSV (1 MOI) for 9 h to assess the effect of UL13 on poly(I:C) or VSV-induced transactivation of the IFN-β promoter. Finally, to evaluate the effects of JSY13 and PRVΔUL13 mutants on the transactivation of the IFN-β promoter, PK15 cells were transfected with the indicated plasmids (the cGAS–STING complex and pcDNA3-Flag-UL13). At 24 h post-transfection, PK15 cells were infected with PRV (0.1 MOI) for 6 and 12 h.

### QRT-PCR and RT-PCR analysis

Total RNAs was isolated using TRIzol (Invitrogen) and reverse transcribed to cDNA using the iScript cDNA Synthesis Kit (BioRad). ISGs and actin were amplified with 2× Taq Master Mix (Vazyme) by semi-quantitative RT-PCR. The mRNA levels of IFN-β and GAPDH were quantitated by SYBR green-based quantitative real-time RT-PCR using a Life Technology instrument. The amplification parameters were 95 ℃ for 30 s, followed by 40 cycles of 95 ℃ for 10 s and 60 ℃ for 30 s. Melting curve analysis was subsequently conducted. Fold changes in mRNA expression were calculated using the ΔΔCT method. The primers used are listed in Table 1.

**Table 1:** **The primer sequences for RT- and QRT-PCR.**

Name	Sequence
PigIFNB1-F	5′-TTGGCATGTCAGAAGCTCCT-3′
PigIFNB1-R	5′-CTGGAATTGTGGTGGTTGCA-3′
PigGAPDH-F	5′-CCTTCATTGACCTCCACTACA-3′
PigGAPDH-R	5′-GATGGCCTTTCCATTGATGAC-3′
CanineIFNB1-F	5′-ACTTCACCTGGGACAACAGG-3′
CanineIFNB1-R	5′-GCTGTACTCCTTGGCCTTCA-3′
CanineGAPDH-F	5′-GGCTGAGAACGGGAAACTTG-3′
CanineGAPDH-R	5′-TCACCCCATTTGATGTTGGC-3′
HumanIFNB1-F	5′-TTCACCAGGGGAAAACTCAT-3′
HumanIFNB1-R	5′-TCCTTGGCCTTCAGGTAATG-3′
HumanGAPDH-F	5′-CCACCCAGAAGACTGTGGAT-3′
HumanGAPDH-R	5′-TTCAGCTCAGGGATGACCTT-3′
ISG15-F	5′-ACTGCATGATGGCATCGGAC-3′
ISG15-R	5′-CAGAACTGGTCAGCTTGCAC-3′
ISG20-F	5′-CAGGATTCCCGGCTTGAAGT-3′
ISG20-R	5′’-CTGGCATCTTCCACCGAGTT-3′
ISG54-F	5′-GCACAGCAATCATGAGTGAGAC-3′
ISG54-R	5′-TTTCCTCCACACTTGAGCCG-3′
ISG56-F	5′-GACCTACGTCTTCCGACACG-3′
ISG56-R	5′-CTTCTGCTTTGCTGTGGTCG-3′
RNase L-F	5′-AAGCGCCATAACAACCCTCA-3′
RNase L-R	5′-GCATGTTCACGTCTGCTCCA-3′
Viperin-F	5′-GCACCTGGACTCTGATTGCT-3′
Viperin-R	5′-TTGGGCAAAACAGCTCATGC-3′
MxA-F	5′-GATCCGGCTCCACTTCCAAA-3′
MxA-R	5′-CTCTTGTCGCTGGTGTCACT-3′
PKR-F	5′-GCACTTCTAGCCATCTGGTCA-3′
PKR-R	5′-GATGTGCTCGTTGTGGGAGA-3′
Actin-F	5′-TGCTGTCCCTGTACGCCTCTG-3′
Actin-R	5′-ATGTCCCGCACGATCTCCC-3′

### CCK-8 assay

Cell proliferation was determined using the CCK-8 assay. PK15 cells were seeded at 6 × 10^4^ cells per well in 24-well plates overnight and then untransfected or transfected with 200 ng of pcDNA3-Flag or pcDNA-Flag-UL13. The proliferative ability of the cells was evaluated 0, 12, 24, and 36 h post transfection. The cells were then digested and seeded into 96-well plates. CCK-8 reagent (10 μL) was added to the wells, and the plates were incubated at 37 °C for 2 h. The optical density of each sample was measured at a wavelength of 450 nm by using an enzyme-labelled instrument (Thermo Fisher Scientific).

### Western blot analysis

Whole-cell extracts were prepared in SDS buffer and subjected to SDS-PAGE and then transferred to a polyvinylidene difluoride (PVDF) membrane (Pall Corp). The membranes were blocked with 3% nonfat milk in phosphate-buffered saline with 0.5% Tween 20 (PBST) for 1 h at room temperature and then incubated with a specific primary antibody overnight at 4 °C followed by incubation with secondary antibodies for 4 h at 4 ℃. The positive bands were visualized using the enhanced ECL reagent.

### Immunofluorescence microscopy

A549 cells were grown on coverslips and transfected with pcDNA3-Flag-UL13, UL13- D194A/V195A, and pcDNA3-Flag. They were subsequently treated with 10 μg/mL poly(I:C) for 9 h. Alternatively, the cells were infected with PRV (0.1 MOI) for 12 h. The cells were fixed and permeabilized with 4% formaldehyde and 0.1% Triton X-100 at 37℃ for 30 min. After washing with glycine-PBS, the cells were blocked with 3% bovine serum albumin (BSA) in PBS for 30 min at 37 ℃. The coverslips were incubated with Flag or the UL42 antibody (1:300) for 1 h and then with the Alexa Fluor 555- or 488-conjugated goat anti-mouse IgG antibody (1:500) for 30 min. After being washed with PBST three times, the coverslips were incubated with the IRF3 antibody (1:100) for 1 h and then with the Alexa Fluor 488- or 555-conjugated goat anti-rabbit IgG antibody (1:500) for 30 min. The slides were stained with DAPI containing the anti-fade DABCO solution (Sigma) after they were washed three times with PBST. Images were captured under a Nikon fluorescence microscope (TS100-F; DSRi2).

### IRF3 dimerization assay

IRF3 dimerization in native PAGE and western blot analysis were conducted as described in a previous study [28]. Briefly, cells in 6-well plates were transfected with pcDNA3-Flag-UL13 or a pcDNA3-Flag empty vector for 24 h. They were then treated with poly(I:C) or infected with VSV (1 MOI) for 9 h. The cells were subsequently lysed with lysis buffer [50 mM Tris (pH 7.5), 150 mM NaCl, 1 mM EDTA and 0.05% NP-40] containing a phosphatase inhibitor cocktail (Thermo). A native gel (8%) was pre-run in a native running buffer for 30 min at 66 V, and the cell lysates were loaded and run at 60 V. Monomeric and dimerized IRF3 molecules were detected by western blot analysis using an anti-IRF3 antibody.

### Coimmunoprecipitation assay

Cells were collected with a lysis buffer supplemented with a phosphatase inhibitor cocktail and incubated with the anti-HA or anti-IRF3 antibody in the presence of Protein A/G agarose (Sigma) for 3 h at 4 ℃. Mouse or rabbit IgG was used as a negative control. The beads were washed 8–10 times with ice-cold lysis buffer. The precipitates were mixed with SDS buffer and boiled for 10 min at 96 ℃. After centrifugation at 6000 rpm for 1 min, the supernatant was collected and used for western blot analysis.

### Chromatin immunoprecipitation assay

The chromatin immunoprecipitation (ChIP) assay was performed as described previously [29]. PK15 cells on 10-cm dishes were transfected with the indicated plasmids or treated with poly(I:C) and then cross-linked with 1% formaldehyde. Cell extracts were sonicated to generate 200-bp to 1000-bp DNA fragments. A portion of cell extracts from each sample was aliquoted as an input sample. Protein–DNA complexes were immunoprecipitated with the anti-HA or anti-IRF3 antibody; mouse or rabbit IgG was used as a negative control. After reverse cross-linking and phenol–chloroform extraction, DNA was purified using a Qiagen plasmid kit. PCR was performed to visualize the enriched DNA fragments. The binding of IRF3 to the IFN-β promoter was detected with the forward primer, 5′-CAG TTC ACT AAA ACT TTA CC-3′, and the reverse primer, 5′-TAT TTA TAC TGG AAG GCC CTC-3′. The primers used to amplify the GAPDH promoter were used in a previous report [30].

### Construction of recombinant virus

PRVΔUL13 or JSY13 with UL13 gene deletion was generated using the CRISPR-Cas9 system. Three small guide RNAs (sgRNA1, sgRNA2, and sgRNA3) were designed and cloned into the PX335 vector. Vero-E6 cells at 50% confluence were transfected with two sgRNAs (ΔUL13-1: sgRNA1 (0.5 μg) and sgRNA3 (0.5 μg); ΔUL13-2: sgRNA2 (0.5 μg) and sgRNA3 (0.5 μg)) and the PRV genome (1 μg) by using a transfection reagent (Mirus Bio LLC). The PRV genome was extracted using the Hirt procedure [31]. The cells were collected when a visible cytopathic effect (CPE) was observed, and the recombinant virus was purified using the plaque formation assay. UL13 deletion and the purity of the recombinant viruses were determined by PCR. The sequences of the small guide RNAs are listed in Table 2.

**Table 2:** **sgRNAs that target UL13 gene in PRV.**

Name	Sequence
sgRNA1-F	5′-CACCGCGCGGCCGCCCATCCACCG-3′
sgRNA1-R	5′-AAACCGGTGGATGGGCGGCCGCGC-3′
sgRNA2-F	5′-CACCGGGCCGCGCGCAGCCGCGCG-3′
sgRNA2-R	5′-AAACCGCGCGGCTGCGCGCGGCCC-3′
sgRNA3-F	5′-CACCGGACATCCTCGAGGAGGAGC-3′
sgRNA3-R	5′-AAACGCTCCTCCTCGAGGATGTCC-3′

### Viral growth curve

Viral growth curve analysis was conducted to compare the growth kinetics of the JSY13 and PRVΔUL13 mutants. Monolayer PK15 cells or MDCK cells infected with the virus (0.1 MOI) were harvested at 0, 2, 4, 6, 12, 18, 24, and 36 h post-infection and then stored at − 80℃. After three freeze–thaw cycles, the viral titre at each time point was determined using plaque formation assay on Vero-E6 cells in triplicate.

### Statistical analysis

All experiments were performed at least three times unless otherwise indicated. Data are presented as the means ± standard deviations (SDs). Statistical significance between groups was determined using Student’s t-test in GraphPad Prism 7.0 software (La Jolla, CA, USA). ^*^P < 0.05, ^**^P < 0.01, ^***^P < 0.001.

## Results

### UL13 blocks IFN-β transactivation mediated by the cGAS–STING pathway

**Figure 1:**
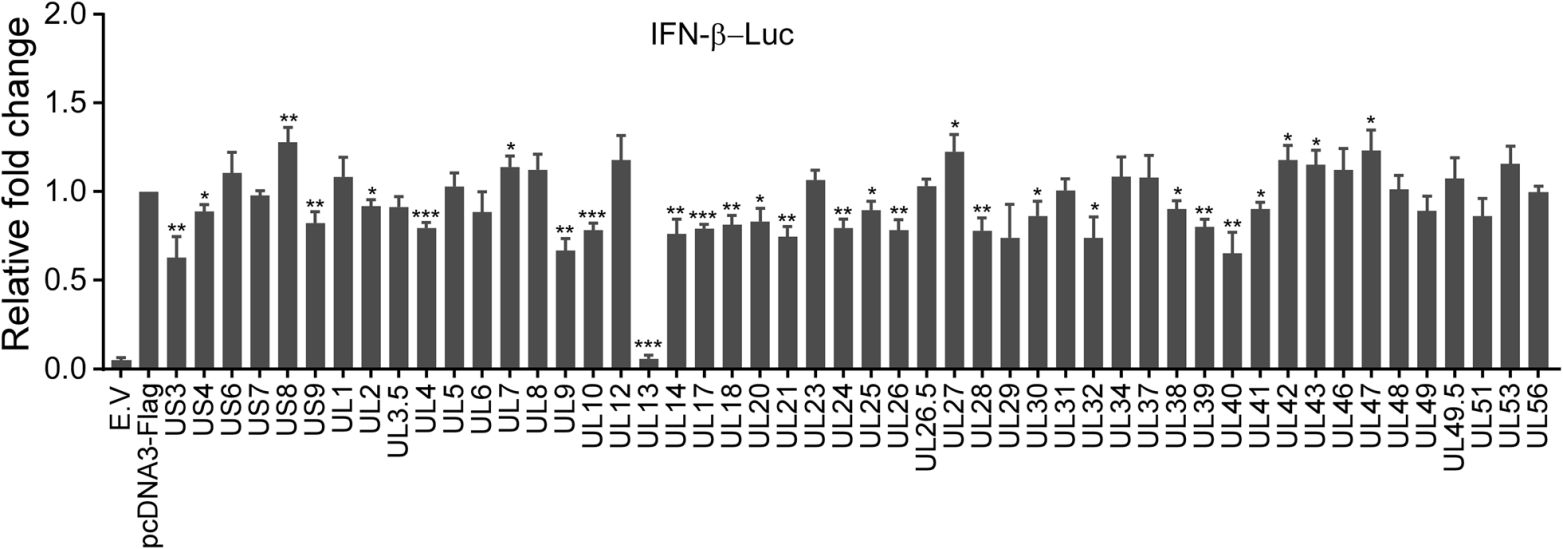
**Screening of PRV open reading frames (ORFs) that modulate the cGAS–STING-induced transactivation of the IFN-β promoter.** PK15 cells were transfected with IFN-β-Luc (200 ng), pCMV-RL (2 ng) mixed with pcDNA4-HA-cGAS (50 ng), pcDNA3-Flag-STING (20 ng), pcDNA3-Flag (200 ng), or pcDNA3-Flag expressing PRV ORFs (200 ng). The total amount of DNA was normalized using the pcDNA3-Flag and pcDNA4-HA empty vectors (E.V) in the negative control. The cells were collected 30 h post-transfection and then analysed for luciferase activity. The fold change in relative luciferase activity is calculated as the luciferase activity induced by cGAS–STING with or without each PRV ORF divided by that induced in the pcDNA3-Flag control group.

To screen PRV ORFs that regulate IFN-β production mediated by the cGAS–STING pathway, 50 PRV ORFs, including 6 US genes and 44 UL genes, were amplified and then cloned into the pcDNA3-Flag expression vector. A dual-luciferase reporter assay was subsequently conducted with the transfection of cGAS, STING, and each PRV ORF. The results showed that UL13 almost abolished cGAS–STING-mediated transactivation of the IFN-β promoter. Eight other ORFs, namely, US3, UL9, UL14, UL21, UL26, UL28, UL32, and UL40, could suppress the cGAS–STING transactivation of the IFN-β promoter by more than 25%. In contrast, six ORFs, namely, US8, UL7, UL27, UL42, UL43, and UL47, markedly enhanced the cGAS–STING-mediated transactivation of the IFN-β promoter (Figure 1). These data suggest that PRV-encoded viral proteins can either activate or inhibit IFN-β promoter activity via the cGAS–STING signalling pathway and that UL13 greatly inhibits IFN-β production.

### UL13 inhibits IFN-β transactivation mediated by cGAS–STING via IRF3 targeting

**Figure 2:**
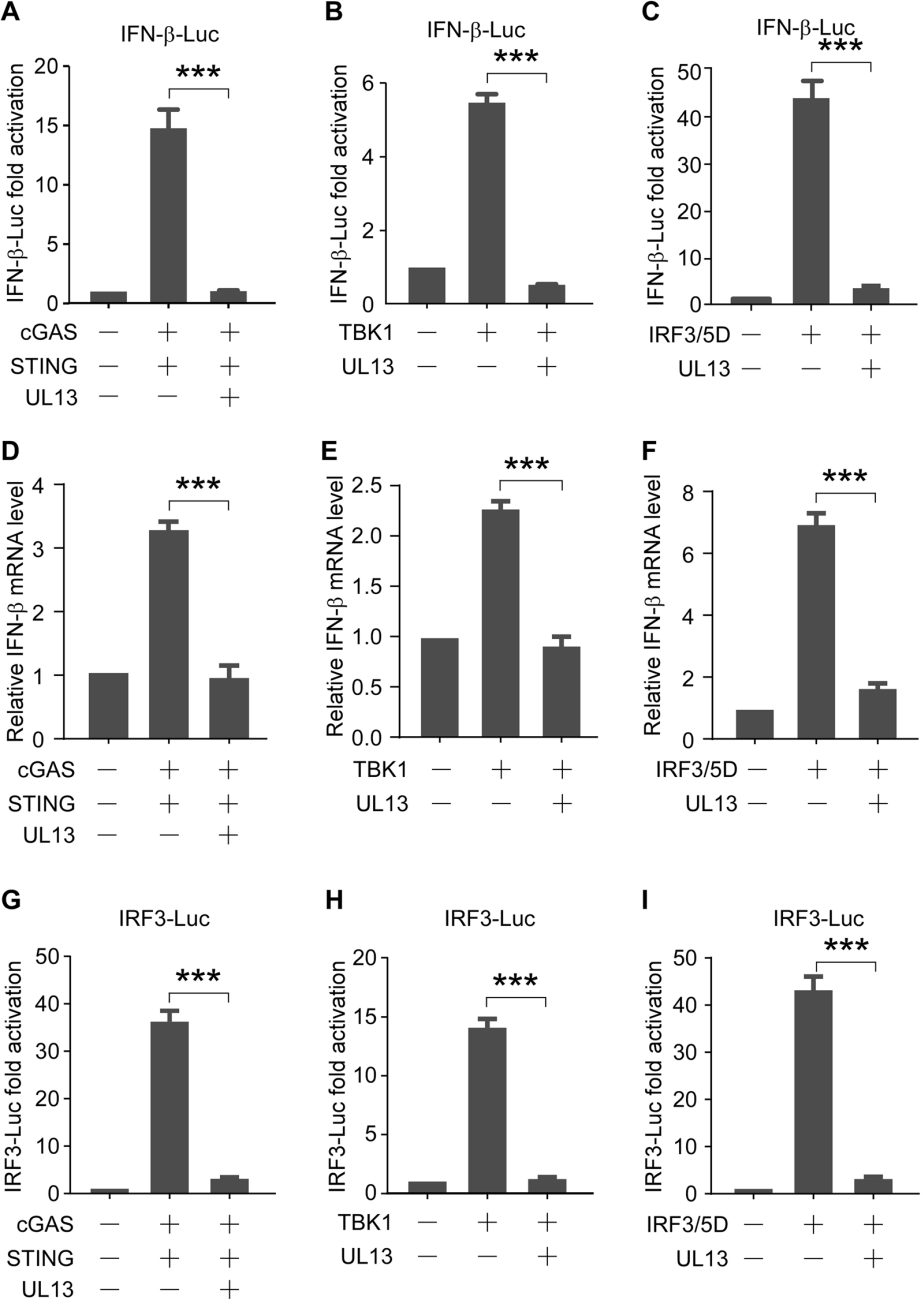
**UL13 suppresses IFN-β transactivation by the cGAS–STING effectors, TBK1, and IRF3.** **A**, **G** PK15 cells were transfected with IFN-β-Luc (200 ng) in (**A**) or IRF3-Luc (200 ng) in (**G**), pCMV-RL (2 ng) mixed with pcDNA4-HA-cGAS (50 ng), pcDNA3-Flag-STING (20 ng), pcDNA3-Flag (200 ng) or pcDNA3-Flag-UL13 (200 ng). The cells were collected 30 h post-transfection and then analysed for luciferase activity. The fold activation of luciferase activity is calculated as the luciferase activity induced by cGAS–STING with or without PRV UL13, divided by that induced by the empty vector. **B**, **H** Dual-luciferase reporter assays were performed as in (**A**, **G**), except that pcDNA4-HA-TBK1 (250 ng) was used instead of pcDNA4-HA-cGAS (50 ng) and pcDNA3-Flag-STING (20 ng). **C**, **I** The dual-luciferase reporter assay was performed as in (**A**, **D**) except that pcDNA4-HA-IRF3/5D (250 ng) was used instead of pcDNA4-HA-cGAS (50 ng) and pcDNA3-Flag-STING (20 ng). **D**–**F** were conducted as in (**A**–**C**), except that transfection was performed without FN-β-Luc and pCMV-RL. The mRNA expression of IFN-β was then analysed by QRT-PCR.

Upon recognition of viral DNA, cGAS leads to cGAMP synthesis; translocation of STING from the endoplasmic reticulum (ER) to the Golgi apparatus; phosphorylation of TBK1, IRF3, IRF7, or NF-κB; and expression of IFNs [32]. To determine the target of UL13 in the blocking of IFN-β production mediated by the cGAS–STING pathway, a dual-luciferase reporter assay was performed to assess the effect of UL13 on TBK1 and IRF3/5D, a constitutively active form of IRF3 containing five C-terminal substitutive Asp (D) residues [33], in addition to the cGAS–STING pathway. We found that cGAS–STING (Figure 2A), TBK1 (Figure 2B), and IRF3/5D (Figure 2C) were able to increase the luciferase activity of IFN-β-Luc, but not in the presence of UL13. In addition, we also checked the mRNA level of the endogenous IFN-β gene, and the results also showed that UL13 could suppress the increase in IFN-β gene expression induced by the cGAS–STING (Figure 2D), TBK1 (Figure 2E), and IRF3/5D (Figure 2F) pathways. To rule out a potential cell type-specific effect, similar experiments were performed on 293T and MDCK cells. Similarly, PRV UL13 suppressed the cGAS–STING-induced transactivation of the IFN-β gene in MDCK (Additional file 1A, B) and 293T cells (Additional file 1C, D). To verify whether IRF3 is a direct target of UL13, IRF3-Luc containing four IRF3 responsive elements of the IFN-β promoter was generated. Similarly, cGAS–STING (Figure 2G), TBK1 (Figure 2H), and IRF3/5D (Figure 2I) were able to increase the luciferase activity of IRF3-Luc but not in the presence of UL13. These data indicate that the cGAS–STING downstream effector IRF3 is a potential target of UL13.

**Figure 3:**
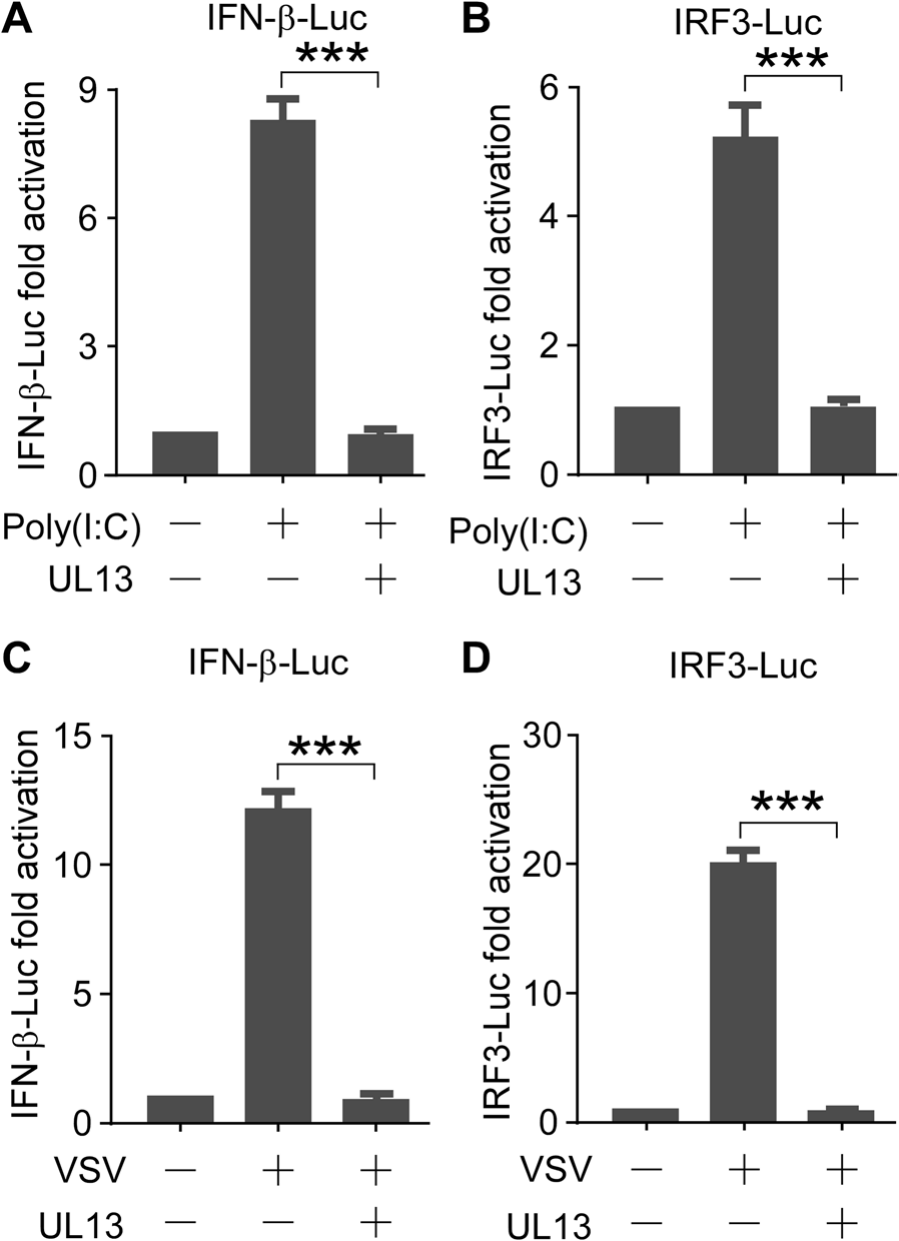
**PRV UL13 inhibits poly(I:C)- or VSV-induced IFN-β transactivation.** **A**, **C** PK15 cells were transfected with IFN-β-Luc (200 ng), pCMV-RL (2 ng) mixed with pcDNA3-Flag (200 ng), or pcDNA3-Flag-UL13 (200 ng) for 24 h; the cells were then treated with poly(I:C) (10 μg/mL) in (**A**) or infected with VSV (1 MOI) in (**C**) for 9 h. The fold activation of luciferase activity is the product of the luciferase activity induced by poly(I:C) with or without PRV UL13 divided by that induced by the empty vector. **B**, **D** The dual-luciferase reporter assays were performed as in (**A**, **C**), except that IRF3-Luc was used instead of IFN-β-Luc.

Previous studies have shown that VSV or poly(I:C) can induce IFN-β by activating the IRF3 signalling pathway [11, 34, 35]. Thus, we also evaluated the effect of UL13 on the VSV- or poly(I:C)-triggered transcriptional induction of the IFN-β gene. A dual-luciferase reporter assay of PK15 cells transfected with IFN-β-Luc, with or without UL13, was performed. Subsequently, the cells were treated with poly(I:C) or VSV infection. We found that both poly(I:C) and VSV increased the luciferase activities of the IFN-β promoter, which were abolished by UL13 (Figure 3A, C). Similarly, the poly(I:C)- or VSV-triggered luciferase activation of IRF3-Luc was suppressed by UL13 (Figure 3B, D). The results of the CCK8 experiment showed that PRV UL13 exerted no effect on cell viability (Additional file 2). Considering the combined findings, we conclude that PRV UL13 inhibits the cGAS–STING-triggered transcriptional activation of the IFN-β gene by targeting IRF3.

### The kinase activity of UL13 is essential for its anti-IFN-β function

**Figure 4:**
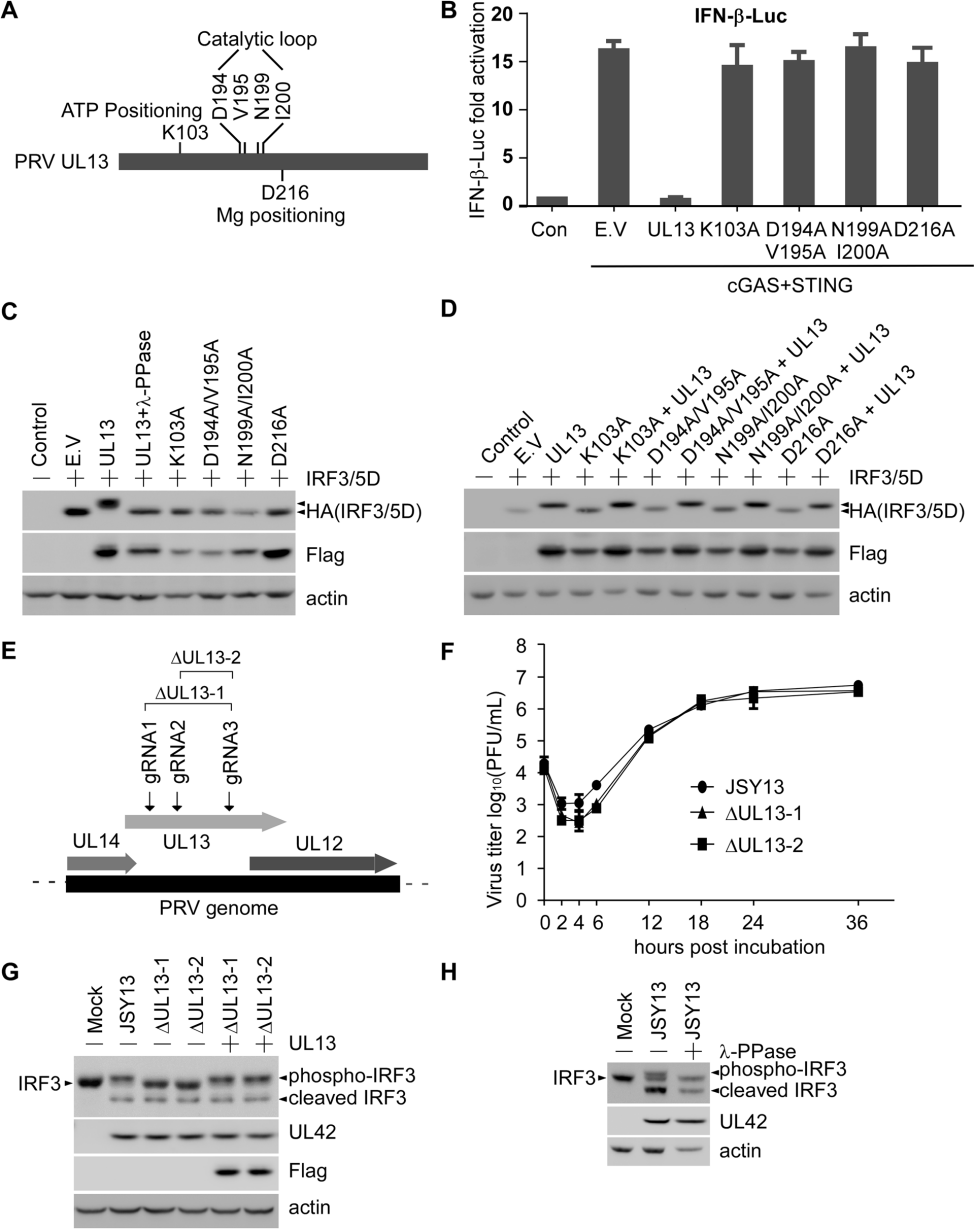
**UL13 kinase activity is essential for inhibiting IRF3 function. A** Schematic presentation of the kinase functional sites of PRV UL13. **B** The dual-luciferase reporter assay was performed as in Figure 1. The fold activation of luciferase activity is the product of the luciferase activity induced by cGAS–STING with or without PRV UL13 or its kinase-dead mutants, divided by that induced by the empty vector. **C** PK15 cells were transfected with pcDNA4-HA or pcDNA4-HA-IRF3/5D mixed with pcDNA3-Flag-UL13, or its kinase-dead mutants for 30 h. Whole cells were collected with lysis buffer, and the lysate of the UL13-transfected group was treated with lambda protein phosphatase. HA-tagged IRF3/5D was detected by anti-HA, and Flag-tagged UL13 and its kinase-dead mutants were detected by anti-Flag. **D** PK15 cells were transfected with pcDNA4-HA or pcDNA4-HA-IRF3/5D mixed with pcDNA3-Flag-UL13, the UL13 kinase-dead mutant or their mixture for 30 h. Whole cells were collected with lysis buffer. HA-tagged IRF3/5D was detected by anti-HA, and Flag-tagged UL13 and its kinase-dead mutants were detected by anti-Flag. **E** Schematic presentation of the location of gRNAs used to generate UL13 deletion mutants. **F** PK15 cells infected with 0.1 MOI JSY13 or PRVΔUL13 mutants were harvested at the indicated time points, and virus titre measurements were conducted using the plaque formation assay. **G** PK15 cells were transfected with or without pcDNA3-Flag-UL13 for 18 h and then infected with 1 MOI of the JSY13 or ΔUL13 mutants for 16 h. Whole cells were collected with lysis buffer. The cell lysates were separated by SDS-PAGE and immunoblotted with an antibody against IRF3, UL42, Flag or actin. **H** PK15 cells were infected with JSY13 (1 MOI) for 16 h. Whole cells were collected with lysis buffer, and the lysate was treated with lambda protein phosphatase. Endogenous IRF3 was detected with the anti-IRF3 antibody.

Given that PRV UL13 is a viral serine/threonine kinase protein, we examined whether the kinase activity is required for its anti-IFN-β function. In accordance with previous findings, we performed amino acid sequence alignment of PRV UL13 with six human herpesviruses (HSV-1, HSV-2, KSHV, Epstein–Barr virus [EBV], varicella-zoster virus [VZV] and infectious laryngotracheitis virus [ILTV]), avian herpesvirus (MDV), the bovine herpesvirus (BHV), and the equine herpesvirus (EHV), to identify residues that are essential for PRV UL13 kinase activity [36–38]. We found that the aforementioned viruses harbour six conserved catalytic residues, namely, K103, D194, V195, N199, I200, and D216. Specifically, K103 is critical for ATP positioning; D194, V195, N199, and I200 are important for catalytic activity; and D216 is essential for Mg^2+^ positioning (Figure 4A). These sites were replaced with alanine, generating four kinase-dead mutants: UL13-K103A, UL13-D194A/V195A, UL13-N199A/I200A, and UL13-D216A. A dual-luciferase reporter assay showed that UL13-mediated suppression of IFN-β-Luc luciferase activity-induced by the cGAS–STING pathway was abrogated by the loss of kinase activity (Figure 4B). These data suggest that UL13 kinase activity is essential for the anti-IFN-β function of UL13.

As shown in Figure 2C, I, IFN-β-Luc and IRF3-Luc activation induced by IRF3/5D, a constitutively active IRF3, is eliminated by UL13. Western blot analysis was performed to further examine whether UL13 regulates IRF3/5D phosphorylation. We found that IRF3/5D migrated more slowly in PK15 cells transiently expressing both IRF3/5D and UL13 than in those expressing IRF3/5D with or without kinase-dead UL13 mutants (Figure 4C). However, this altered migration rate of IRF3/5D by UL13 was restored upon treatment with lambda protein phosphatase (λ-PPase) (Figure 4C). We also conducted a rescue experiment by cotransfecting UL13 and its kinase-dead mutants, and the result showed that IRF3/5D exhibited a reduced migration pattern upon UL13 overexpression (Figure 4D).

To further explore whether UL13 could phosphorylate endogenous IRF3 during PRV infection, two UL13-deleted PRV mutants, designated ΔUL13-1 and ΔUL13-2, were generated using the CRISPR–Cas9 system. UL13 was deleted from each virus using a pair of small guide RNAs specifically targeting the N- and C-termini of the UL13 ORF (Figure 4E). The virus growth curve was analysed to compare the growth kinetics of the JSY13 and ΔUL13 mutants. The results indicated that UL13 deletion slightly impaired virus propagation within 12 h post-infection. However, no substantial difference was observed between the JSY13 and PRVΔUL13 mutants at 12–36 h post-infection (Figure 4F). Similar growth curves of the viruses in MDCK cells were generated (Additional file 3). To determine whether UL13 can phosphorylate endogenous IRF3, PK15 cells were infected with 1 MOI of each of JSY13, ΔUL13-1, and ΔUL13-2 or rescued by transfection with Flag-UL13 and then infected with ΔUL13-1 and ΔUL13-2. The results showed that JSY13 and the two rescued groups showed a slow migrated band (Figure 4G). To determine whether the slow migrated band was phosphorylated IRF3, the JSY13-infected samples were treated with λ-PPase, and the result showed that after treatment the migrated band was then restored to the normal IRF3 band (Figure 4H). Notably, in addition to the phosphorylated IRF3 band, a band smaller than the normal IRF3 was found. This finding suggests that this band might be a cleaved IRF3 (Figure 4H). These data together indicate that PRV UL13 potentially phosphorylates IRF3 and that such a modification negatively modulates IRF3 transcriptional activity.

### UL13 inhibits IRF3 binding to the IRF3-responsive promoter and downstream ISG expression

**Figure 5:**
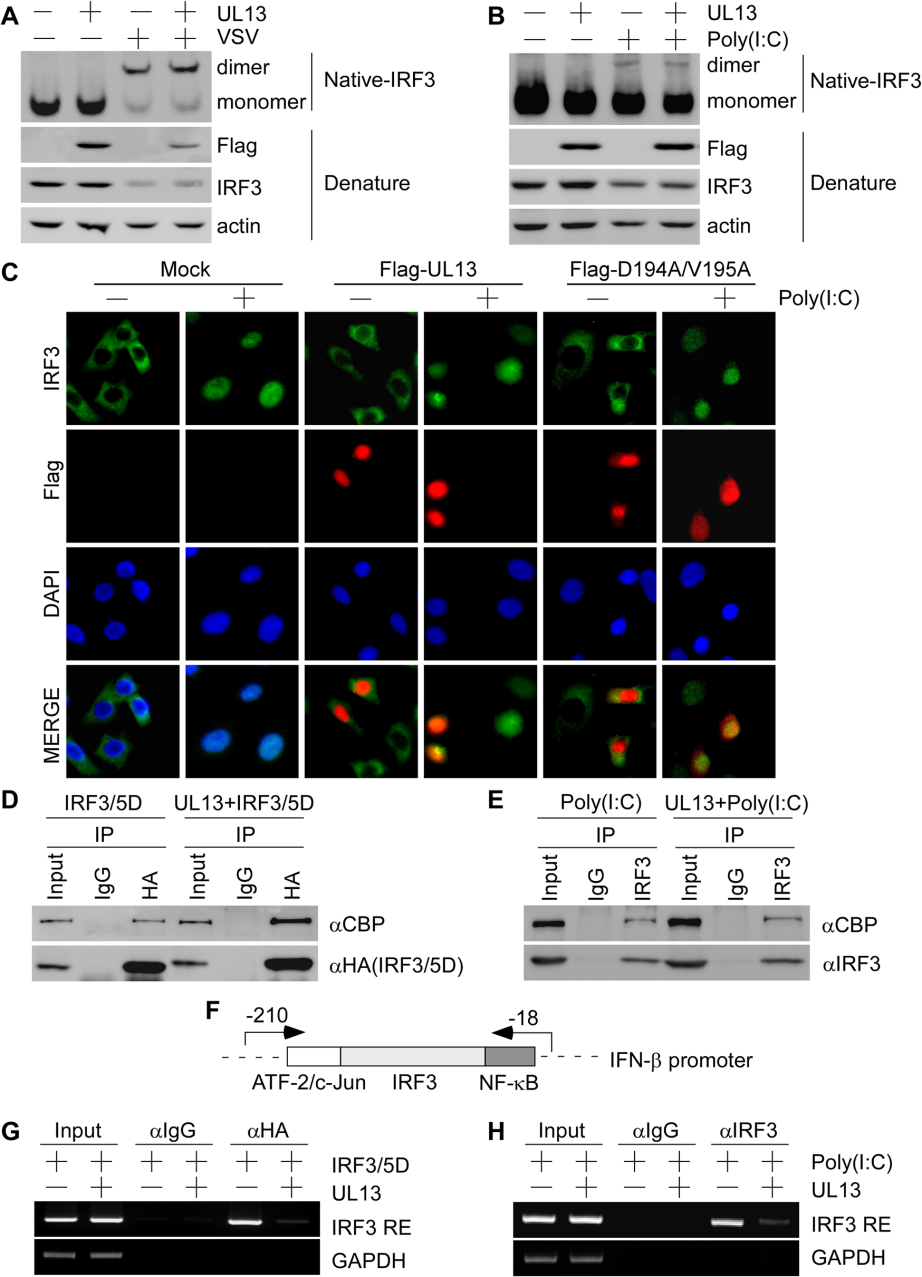
**PRV UL13 inhibits IRF3 binding to IRF3-responsive promoter sequences. A**, **B** PK15 cells were transfected with pcDNA3-Flag (1 μg) or pcDNA3-Flag-UL13 (1 μg) for 24 h and then infected with VSV (1 MOI) (**A**) or treated with poly(I:C) (10 μg/mL) (**B**) for 9 h. Whole-cell extracts were subjected to native PAGE. Dimers or monomers of IRF3, UL13, and actin were detected with the respective antibodies. **C** A549 cells were transfected with pcDNA3-Flag-UL13 or D194A/V195A for 24 h. The transfected cells were treated with poly(I:C) (10 μg/mL) for 9 h. The subcellular location of IRF3 was detected with IRF3 antibody, and UL13 was detected with the Flag antibody. The nuclei were stained with DAPI. **D** PK15 cells were transfected with pcDNA4-HA-IRF3/5D (2 μg) mixed with pcDNA3-Flag or pcDNA3-Flag-UL13 (1 μg) for 30 h. Whole-cell lysates were immunoprecipitated with the HA antibody or control IgG. The immunocomplexes were then used to detect CBP and IRF3/5D with their respective antibodies. **E** PK15 cells were transfected with pcDNA3-Flag or pcDNA3-Flag-UL13 (2 μg) for 30 h and then treated with poly(I:C) (10 μg/mL) for 9 h. Whole-cell lysates were immunoprecipitated with the IRF3 antibody or control IgG. The immunocomplexes were then used to detect CBP and IRF3 with their respective antibodies. **F** Schematic presentation of the IFN-β promoter with the location of IRF3-responsive elements and ChIP primers. **G** PK15 cells were transfected with pcDNA4-HA-IRF3/5D (5 μg) mixed with pcDNA3-Flag or pcDNA3-Flag-UL13 (2 μg) for 30 h. Chromatin was then immunoprecipitated with the HA antibody or control IgG. The binding of IRF3-responsive elements to the IFN-β promoter was quantified by PCR. **H** PK15 cells were transfected with pcDNA3-Flag or pcDNA3-Flag-UL13 (4 μg) for 24 h and then treated with poly(I:C) (10 μg/mL) for 9 h. Chromatin was then immunoprecipitated with the IRF3 antibody or control IgG. The binding of IRF3 to IRF3-responsive elements on the IFN-β promoter was quantified by PCR.

The activation mechanisms of IRF3 as a transcription factor consist of C-terminal phosphorylation, dimerization, nuclear translocation, association with the co-activator CBP/p300, and binding to its responsive elements on the IFN gene promoter [39]. To characterize the regulatory mechanism by which UL13 modulates IRF3 function, we evaluated the effect of UL13 on IRF3 dimerization, nuclear translocation, association with the transcriptional co-activator CBP/p300, or binding to the IFN-β promoter. IRF3 dimer formation was analysed by native PAGE analysis using PK15 cells transiently expressing UL13, followed by VSV infection or poly(I:C) treatment. The results showed that PRV UL13 could not affect the IRF3 dimerization induced by VSV or poly(I:C) (Figure 5A, B). Immunofluorescence assays were then performed to investigate whether UL13 affects poly(I:C)-triggered IRF3 nuclear translocation. The results showed that both UL13 and its kinase-dead mutant (D194A/V195A) exerted no effect on the nuclear translocation of IRF3 (Figure 5C). These results demonstrate that poly(I:C) stimulation activates IRF3 dimerization and nuclear translocation. Thus, we investigated the association of IRF3 with the coactivator CBP/p300. The interaction of IRF3 with CBP/p300 was analysed by a coimmunoprecipitation assay using PK15 cells transiently expressing IRF3/5D alone or with UL13. PRV UL13 could not inhibit the interaction between IRF3/5D and CBP (Figure 5D). To evaluate the effect of UL13 on the interaction between endogenous IRF3 and CBP/p300, we performed coimmunoprecipitation assay using PK15 cells transiently expressing UL13 alone or with poly(I:C) treatment; PRV UL13 exerted no effect on the association between endogenous IRF3 and CBP (Figure 5E). Thus PRV UL13 likely did not alter the nuclear translocation of IRF3 because the interaction between IRF3 and CBP occurred inside the nucleus. Finally, to examine whether UL13 could regulate the binding of IRF3 to IRF3-responsive elements on the IFN-β promoter, a ChIP assay was performed using the primers shown in Figure 5F. The results showed that the binding of IRF3/5D to the IFN-β promoter was evidently attenuated by PRV UL13 (Figure 5G). Similarly, the enriched binding of endogenous IRF3 to the IFN-β promoter induced by poly(I:C) was markedly reduced by UL13 (Figure 5H). The combined data suggest that UL13-mediated IRF3 phosphorylation decreases the binding affinity of IRF3 to the IFN-β promoter.

**Figure 6:**
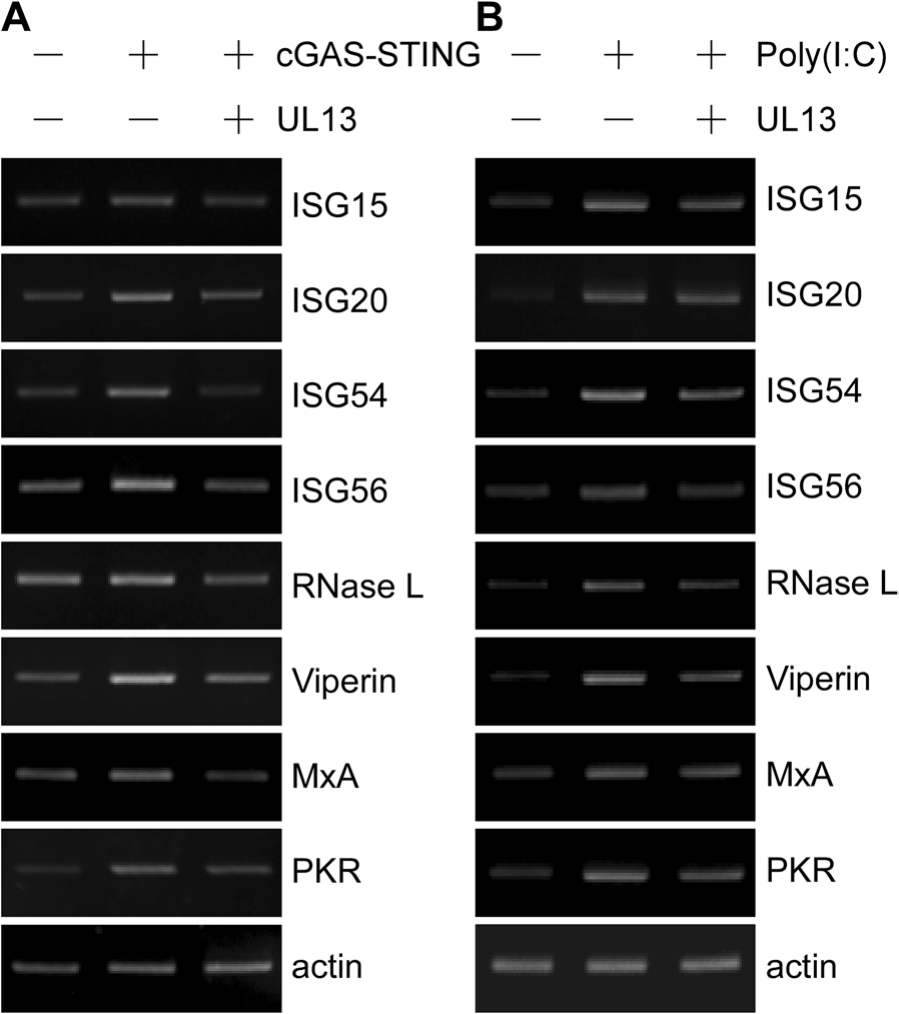
**UL13 suppresses the expression of ISGs induced by cGAS–STING or poly(I:C). A** PK15 cells were transfected with a mixture of pcDNA4-HA-cGAS (50 ng) and pcDNA3-Flag-STING (20 ng), together with pcDNA3-Flag or pcDNA3-Flag-UL13 (200 ng), for 30 h. The total amount of DNA was normalized to the corresponding empty vectors. The mRNA expression levels of ISGs were determined by semi-quantitative RT-PCR. **B** The experiment was performed as in (**A**), except that the PK15 cells were transfected with pcDNA3-Flag or pcDNA3-Flag-UL13 (500 ng) for 24 h, and then either treated with poly(I:C) (10 μg/mL) for 9 h or not.

Once activated, IFN-β binds to IFN receptors and then initiates the cascades of phosphorylation events of Janus kinase (JAK) as well as signal transducer and activator of transcription (STAT) proteins to form an activated STAT complex, which binds to IFN-stimulated response elements (ISREs) in the promoters of ISGs [40]. This process leads to the expression of ISGs that protect hosts against viral infection directly or enhance the host immune response indirectly. A viral life cycle includes entry into host target cells, uncoating, genome translation-replication, and release of mature virions; each of these steps can be targeted by ISGs [41–46]. One study found that porcine ISG15 was upregulated in the early stages of PRV infection and that overexpression of ISG15 efficiently inhibited PRV replication [47]. To test the possible effect of PRV UL13 on ISGs induced by the cGAS–STING pathway or poly(I:C), semi-quantitative RT-PCR was performed. The result showed that the expression levels of multiple ISGs namely, ISG15, ISG20, ISG54, ISG56, RNase L, Viperin, MxA, and PKR were upregulated in PK15 cells transiently transfected with cGAS–STING or treated with poly(I:C), but not in PK15 cells concomitantly transfected with UL13 (Figure 6A, B).

### UL13 deficiency potentiates PRV-triggered transactivation of the IFN-β gene

**Figure 7:**
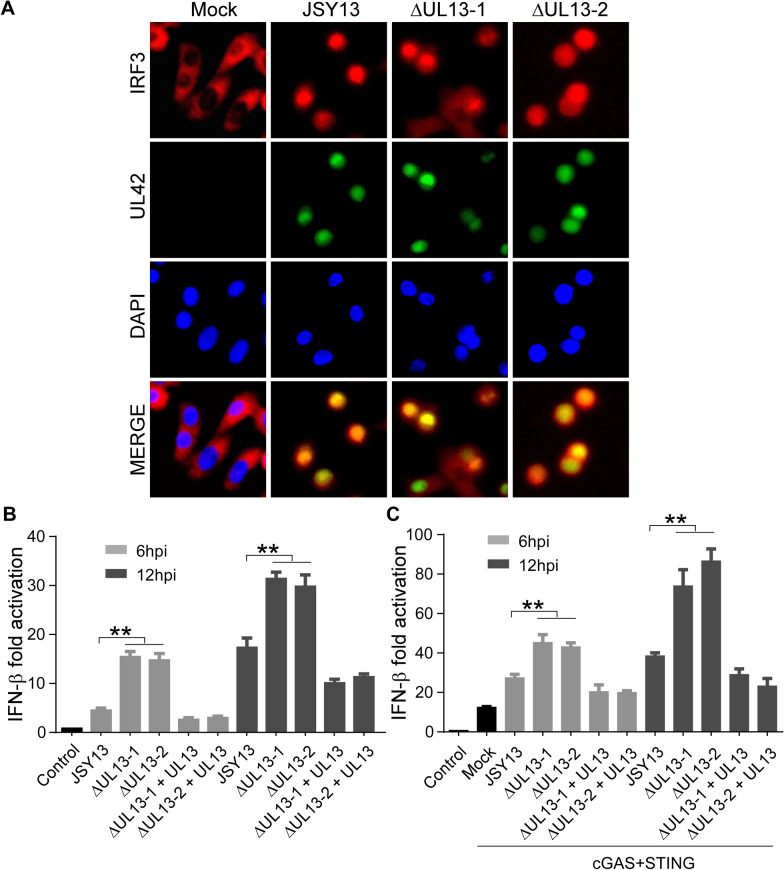
**UL13 deficiency enhances the IRF3-responsive activity of the IFN-β promoter. A** A549 cells were infected with 0.1 MOI JSY13 or its ΔUL13 mutants for 12 h. The cells were fixed and stained with UL42 and IRF3 antibodies. The nuclei were then stained with DAPI. **B** PK15 cells were transfected with IFN-β-Luc (200 ng) and pCMV-RL (2 ng), with or without pcDNA3-Flag-UL13 (100 ng) for 24 h; they were subsequently infected with JSY13 or PRVΔUL13 mutants (0.1 MOI). The cell lysates were collected 6 or 12 h post-infection and then analysed for luciferase activity. The fold activation of luciferase activity is calculated as the luciferase activity induced by the JSY13 and PRVΔUL13 mutants divided by that induced by the empty vector. **C** The dual-luciferase activity assay was performed as in (**B**) except that the PK15 cells were transfected with IFN-β-Luc (200 ng), pCMV-RL (2 ng), pcDNA4-HA-cGAS (50 ng), or pcDNA3-Flag-STING (20 ng), with or without pcDNA3-Flag-UL13 (100 ng).

As shown in Figure 4C, G, we demonstrated that IRF3 could be phosphorylated by UL13. We also showed that UL13 kinase activity was not required for the nuclear translocation of IRF3. Thus, we can speculate that the nuclear translocation of IRF3 could occur via PRV infection and that IRF3 transcriptional activity could subsequently be suppressed by UL13-mediated phosphorylation. To verify this hypothesis, an immunofluorescence experiment was performed to determine the subcellular localization of IRF3 in JSY13 and ΔUL13 mutants. We found that upon JSY13 and ΔUL13 mutant infection, IRF3 nuclear translocation could occurred (Figure 7A). These data suggest that UL13 does not block the PRV-induced nuclear translocation of IRF3. We subsequently examined whether UL13 affects IRF3-mediated IFN-β expression, which was measured using the IFN-β luciferase reporter assay. A dual-luciferase reporter assay in the absence of cGAS–STING showed that JSY13 infection increased the transcriptional activity of the IFN-β promoter, which was further enhanced by PRVΔUL13 mutants at 6 and 12 h post-infection (Figure 7B). However, the transcriptional activity of the IFN-β promoter was rescued by the reintroduction of UL13 into PRVΔUL13-infected cells (Figure 7B). Similarly, a dual-luciferase reporter assay in the presence of cGAS–STING showed that both the JSY13 and PRVΔUL13 mutants could further enhance IFN-β luciferase activity induced by the cGAS–STING complex; moreover, PRVΔUL13 mutants induced enhanced IFN-β-Luc activation relative to that in JSY13 but not in the UL13 rescued group (Figure 7C). These combined data suggest that PRV UL13 phosphorylates IRF3 and subsequently suppresses IRF3-mediated IFN-β transactivation.

## Discussion

Upon recognition of specific molecular components of viruses, the host cell activates multiple signalling cascades that stimulate an innate antiviral response, disrupting viral replication. However, viruses have evolved to develop various strategies to antagonize cellular innate immunity, such as the modification of cytosolic and nuclear signalling effectors by phosphorylation, de-phosphorylation, or ubiquitination [2]. The cGAS–STING DNA sensing pathway has previously been reported to detect PRV infection and activate downstream TBK1 and IRF3 proteins. This process leads to IFN-β production; however, it conversely hydrolyses cGAMP by ectonucleotide pyrophosphatase phosphodiesterase 1 (ENPP1) in PRV-infected and cGAMP-transfected cells, as well as inhibits IRF3 phosphorylation and IFN-β secretion [48]. Whether PRV could evade the cGAS–STING pathway through other mechanisms, particularly via the direct involvement of viral factors, has yet to be determined. Accordingly, we screened 50 PRV ORFs and found that PRV UL13, a serine/threonine protein kinase, could abolish the transcriptional induction of the IFN-β gene mediated by the cGAS–STING pathway.

Phosphorylation, one of the most extensively investigated posttranslational modifications (PTMs), plays a critical role in modulating various biological functions, including the DNA damage response, cell proliferation, programmed cell death, and immune response. Activation of the innate immune response requires the phosphorylation of critical effectors by multiple kinase cascades, which can be used by viruses to create a suitable cellular environment conducive to replication [49]. Previous reports have shown that every herpesvirus encodes one or two kinase proteins that can phosphorylate both viral and cellular proteins to evade the host immune response. IRF3, a key regulator of IFN-β, can be phosphorylated by cellular and viral proteins at multiple phosphorylation sites, leading to either the activation or suppression of IRF3 transcriptional activities and consequently, to increased or decreased IFN-β production. IRF3 activation can be modulated by viral kinase proteins through different processes, including IRF3 dimerization, nuclear localization, complex formation with CBP/p300, and binding to target gene promoters [40]. For instance, VZV ORF47 attenuates the transcriptional activation of the IFN-β gene by inhibiting IRF3 dimerization [33]. The HSV-1 US3-induced hyper-phosphorylation of IRF3 at Ser175 abolishes IRF3 dimerization and nuclear translocation [50]. Feline herpesvirus 1 US3 blocks IFN-β production mediated by the cGAS–STING pathway by inhibiting IRF3 dimerization in a kinase-independent manner [28]. MHV-68 ORF36 suppresses IFN-β by inhibiting the interaction between IRF3 and the transcriptional co-activator CBP [51]. These reports suggest that herpesvirus viral kinase proteins play a conserved role in suppressing IFN-β production but through different mechanisms.

In the current study, we showed that overexpression of PRV UL13 inhibited cGAS–STING-, TBK1-, IRF3/5D-, poly(I:C)-, or VSV-induced transactivation of the IFN-β promoter; moreover, UL13 deletion enhanced the PRV infection-induced transactivation of the IFN-β promoter. Notably, PRV UL13 did not alter IRF3 dimer formation, nuclear translocation, or association with the CBP coactivator but markedly weakened IRF3 binding to the IRF3-responsive promoter. Previous studies reported that several conserved herpes virus-encoded kinases may contribute to the anti-IFN function by inhibiting the IRF3 pathway: HSV-1 UL13, HCMV UL97, MHV-68 ORF36, and the EBV BGLF4 kinase protein [51]. However, the specifically evolved mechanisms of the anti-IFN function differed. For instance, MHV-68 ORF36 can inhibit the binding of activated IRF3 to CBP, and EBV BGLF4 suppresses the amount of active IRF3 recruited to the IRF3-responsive element containing the IFN-β promoter region via IRF3 phosphorylation [51, 52]. The anti-IFN function of PRV UL13 seems similar to that of BGLF4. Thus, the identification of UL13-mediated phosphorylation sites in IRF3 deserves further investigation.

PRV UL13 is a virion-associated kinase protein expressed in the early stages of the lytic cycle and targets several viral and cellular substrates [53]. By comparing the PRV UL13 amino acid sequence with those of other herpesviruses, we identified six conserved sites essential for its kinase activity: K103 for ATP positioning, D216 for Mg^2+^ positioning, and D194, V195, N199, and I200 for catalytic activity. We showed that four kinase-dead mutants lost their inhibitory effect on the cGAS–STING-induced transcriptional induction of the IFN-β gene. This finding represents the first evidence demonstrating the kinase catalytic sites of PRV UL13. The UL13-deleted PRV strain was previously reported to increase the mean survival time and moderately decrease neuroinvasion and neurovirulence [54]. However, the underlying mechanism has not been explored. In the present study, we demonstrated that PRV UL13 was necessary for PRV to evade host innate immunity activated by the cGAS–STING DNA sensing pathway, providing a potential explanation. Notably, among the 50 ORFs tested in this study, eight ORFs were able to suppress the cGAS–STING-mediated transactivation of the IFN-β promoter by more than 25%, and six ORFs seemed to play an opposite role in this process. This finding indicates that during PRV infection, intricate regulation of cGAS–STING signalling may be pivotal for fulfilling the conditions required for virus replication.

In summary, this study was the first to demonstrate that PRV UL13 effectively inhibits cGAS–STING-mediated IFN-β production by phosphorylating activated IRF3 and disrupting IRF3 binding to the IRF3-responsive promoter. This study provides new insights into innate immune evasion and the establishment of persistent infection by PRV.

## Supplementary information

**Additional file 1. UL13 suppresses IFN-β transactivation induced by the cGAS–STING pathway in MDCK and 293T cells.** (**A**) MDCK cells were transfected with IFN-β-Luc (200 ng) and pCMV-RL (2 ng) along with a mixture of pcDNA4-HA-cGAS (50 ng), pcDNA3-Flag-STING (20 ng), pcDNA3-Flag (200 ng) or pcDNA3-Flag-UL13 (200 ng). The cells were collected 30 hours post-transfection and then analysed for luciferase activity. The fold activation of luciferase activity is calculated as the luciferase activity induced by cGAS–STING with or without PRV UL13, divided by that induced by the empty vector. (**B**) MDCK cells were transfected with pcDNA4-HA-cGAS (50 ng), pcDNA3-Flag-STING (20 ng), pcDNA3-Flag (200 ng), or pcDNA3-Flag-UL13 (200 ng). The cells were collected 30 h post-transfection, and the mRNA level of IFN-β was analysed by QRT-PCR. (**C**) and (**D**) Dual-luciferase reporter assays and QRT-PCR were performed in 293T cells as in (A) and (B), respectively.

**Additional file 2. UL13 kinase does not affect cell viability.** PK15 cells were transfected with 200 ng of pcDNA3-Flag-UL13 or empty vector in 24-well plates. Cell viability of PK15 cells was determined using the CCK-8 reagent at 0, 12, 24, 36, and 48 h post-transfection.

**Additional file 3. Growth curve of JSY13 and two UL13 deletion mutants in MDCK cells.** MDCK cells in 6-well plate were infected with JSY13 (0.1 MOI) or ΔUL13 mutants (0.1 MOI). Virus-infected samples were collected 0, 2, 4, 6, 12, 18, 24, and 36 h post-incubation. Virus titre measurements were conducted using the plaque formation assay.

## Data Availability

All data generated or analysed during this study are included in this published article.

## Abbreviations

CBP: CREB-binding protein
cGAS: cyclic GMP-AMP (cGAMP) synthase
DMEM: Dulbecco’s modified Eagle medium
HSV-1: herpes simplex virus 1
IFN-β: interferon-β
ISG: interferon stimulated gene
KSHV: Kaposi’s sarcoma-associated herpesvirus
MDCK: Madin–Darby canine kidney cell-line
ORF: open reading frame
poly(I:C): polyinosinic:polycytidylic acid
PRV: pseudorabies virus
STING: stimulator of interferon genes
TBK1: TANK-binding kinase 1
VSV: vesicular stomatitis virus
VZV: varicella-zoster virus
