# Supplementary figures and images for: PRV UL13 inhibits cGAS–STING-mediated IFN-β production by phosphorylating IRF3
Source: Vet Res. 2020 Sep 15;51:118. doi: 10.1186/s13567-020-00843-4 (PMC7493860; doi:10.1186/s13567-020-00843-4)

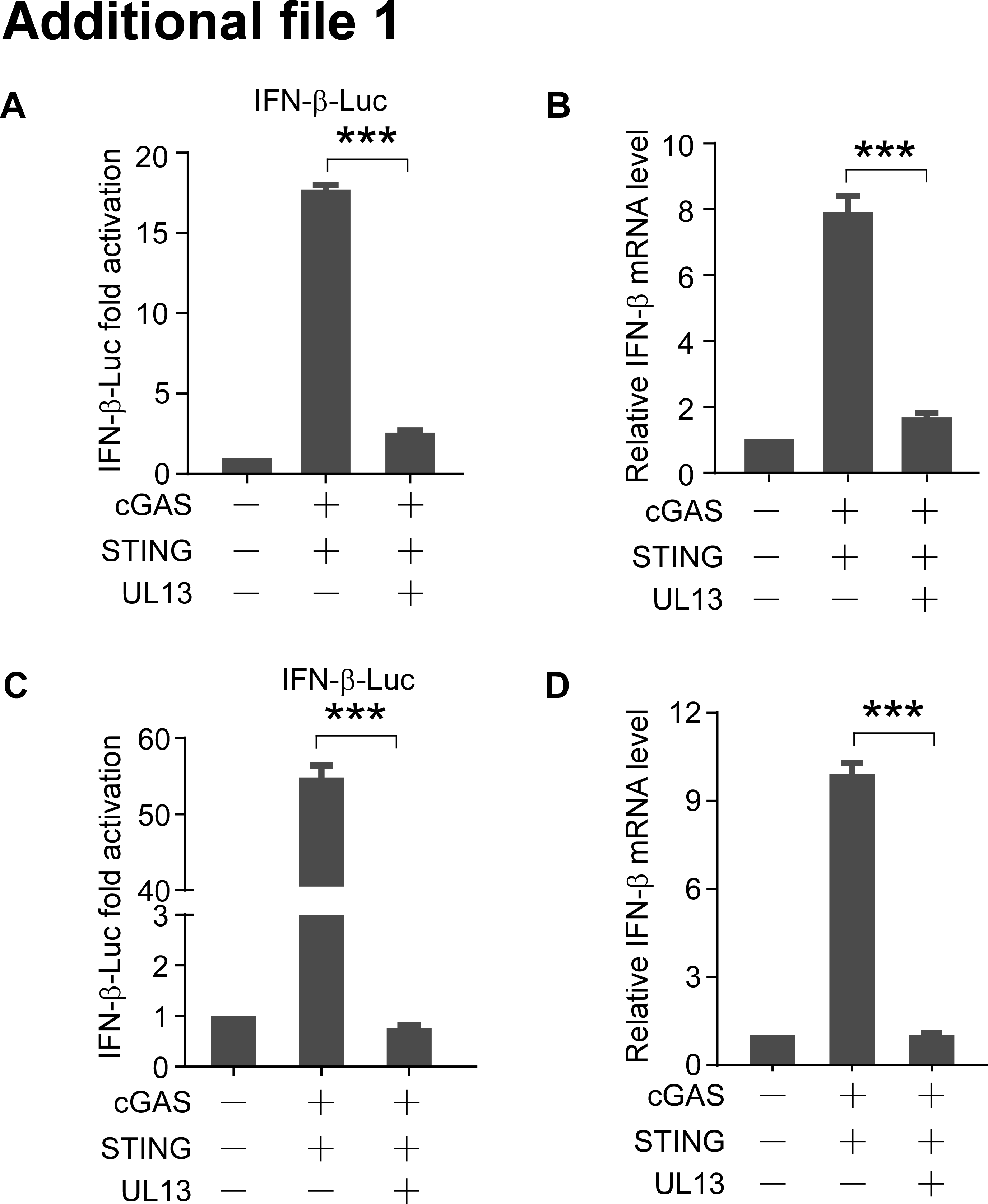

Supplement: Supplementary file 1 — Additional file 1. UL13 suppresses IFN-β transactivation induced by the cGAS–STING pathway in MDCK and 293T cells. (A) MDCK cells were transfected with IFN-β-Luc (200 ng) and pCMV-RL (2 ng) along with a mixture of pcDNA4-HA-cGAS (50 ng), pcDNA3-Flag-STING (20 ng), pcDNA3-Flag (200 ng) or pcDNA3-Flag-UL13 (200 ng). The cells were collected 30 hours post-transfection and then analysed for luciferase activity. The fold activation of luciferase activity is calculated as the luciferase activity induced by cGAS–STING with or without PRV UL13, divided by that induced by the empty vector. (B) MDCK cells were transfected with pcDNA4-HA-cGAS (50 ng), pcDNA3-Flag-STING (20 ng), pcDNA3-Flag (200 ng), or pcDNA3-Flag-UL13 (200 ng). The cells were collected 30 h post-transfection, and the mRNA level of IFN-β was analysed by QRT-PCR. (C) and (D) Dual-luciferase reporter assays and QRT-PCR were performed in 293T cells as in (A) and (B), respectively. [file 13567_2020_843_MOESM1_ESM.tif]

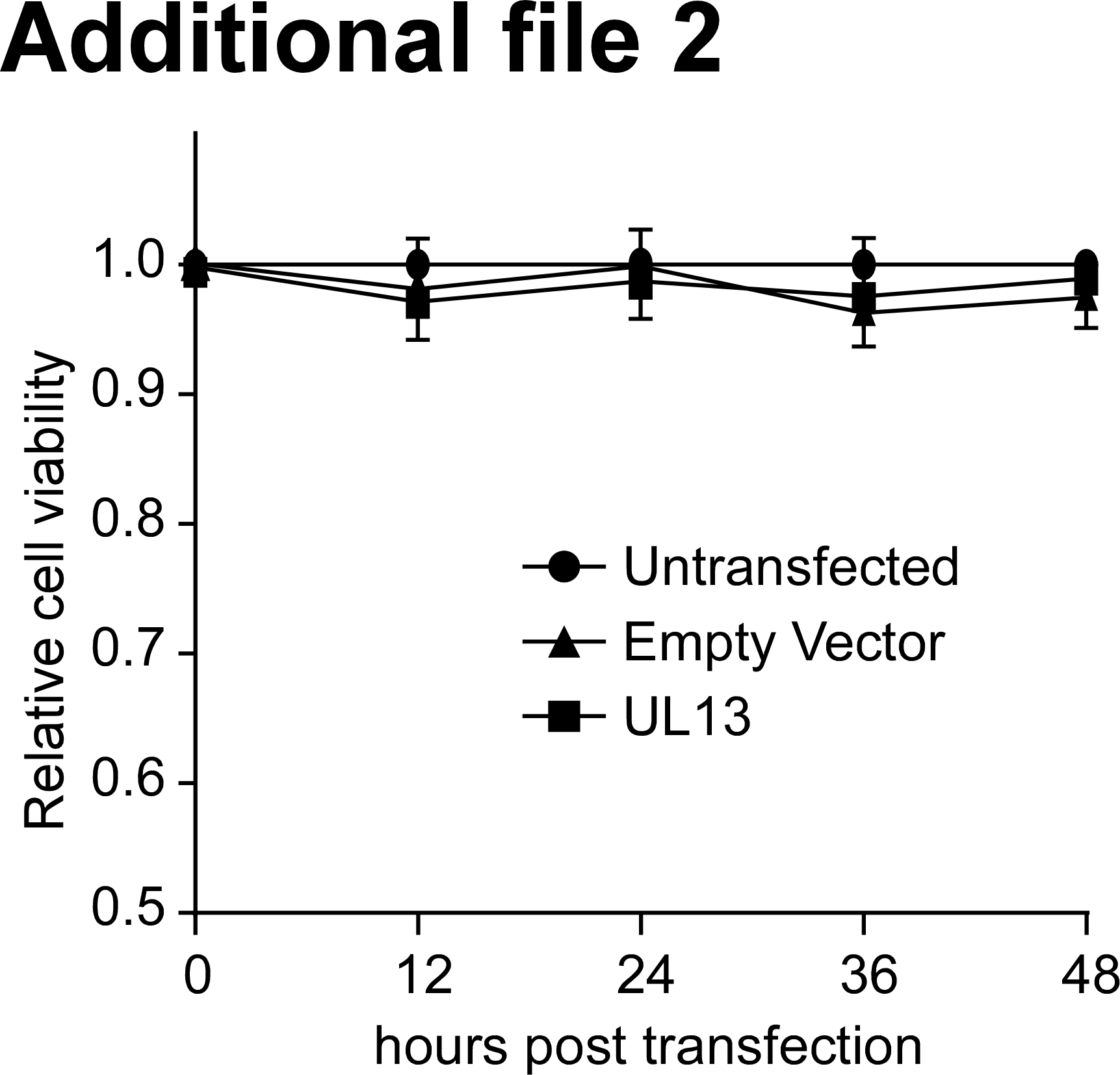

Supplement: Supplementary file 2 — Additional file 2. UL13 kinase does not affect cell viability. PK15 cells were transfected with 200 ng of pcDNA3-Flag-UL13 or empty vector in 24-well plates. Cell viability of PK15 cells was determined using the CCK-8 reagent at 0, 12, 24, 36, and 48 h post-transfection. [file 13567_2020_843_MOESM2_ESM.tif]

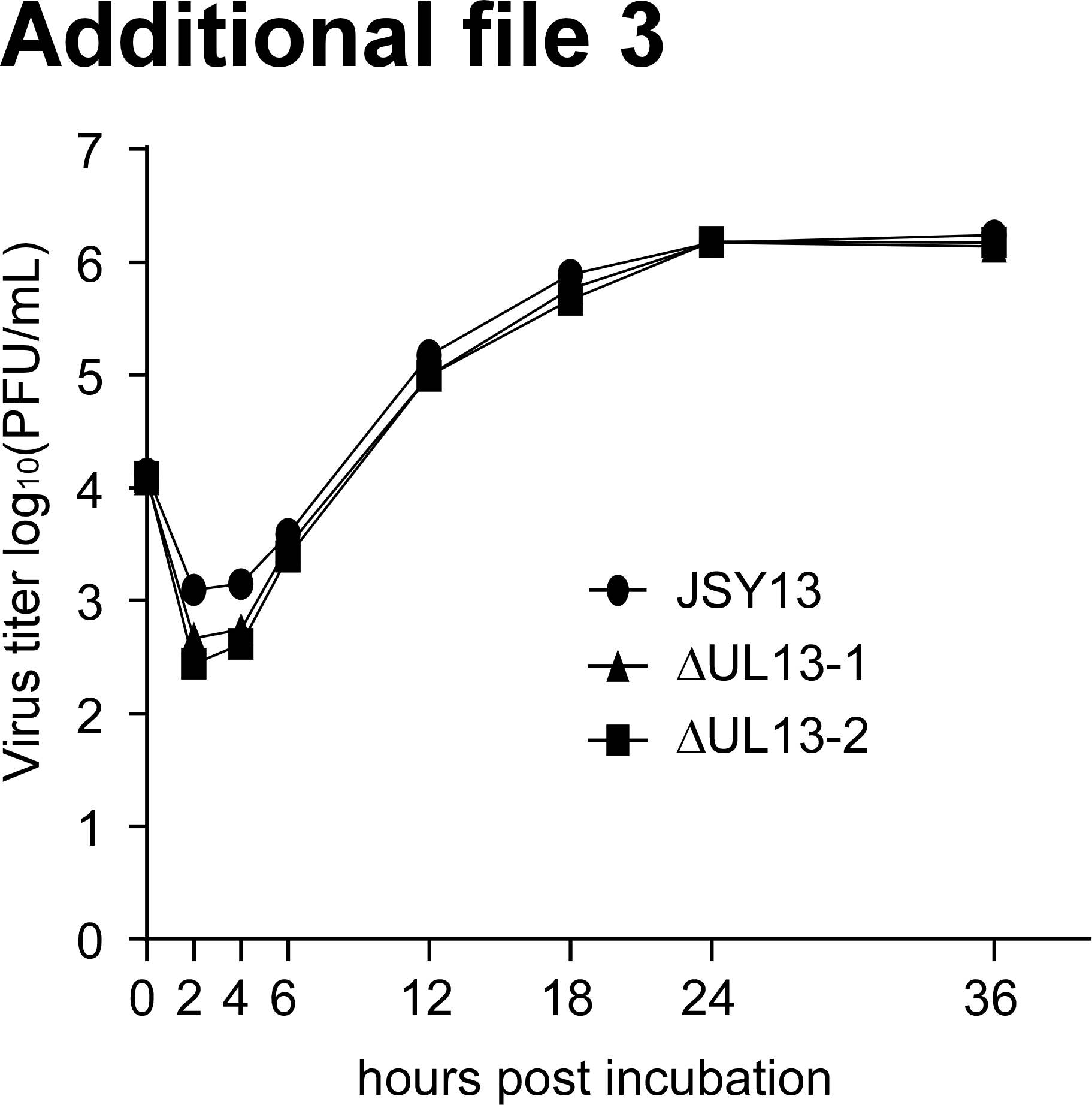

Supplement: Supplementary file 3 — Additional file 3. Growth curve of JSY13 and two UL13 deletion mutants in MDCK cells. MDCK cells in 6-well plate were infected with JSY13 (0.1 MOI) or ΔUL13 mutants (0.1 MOI). Virus-infected samples were collected 0, 2, 4, 6, 12, 18, 24, and 36 h post-incubation. Virus titre measurements were conducted using the plaque formation assay. [file 13567_2020_843_MOESM3_ESM.tif]
